# A Case Report of Good’s Syndrome Diagnosed After Thymomectomy

**DOI:** 10.7759/cureus.85545

**Published:** 2025-06-08

**Authors:** Muhammad Izzul Hazim Ahmad, Chee Yik Chang

**Affiliations:** 1 Internal Medicine, Hospital Sultanah Aminah, Johor Bahru, MYS; 2 Infectious Diseases, Hospital Sultanah Aminah, Johor Bahru, MYS

**Keywords:** good's syndrome, hypogammaglobulinemia, microsporidium spp, salmonella spp, thymomectomy

## Abstract

Good's syndrome is a rare immunodeficiency disorder associated with thymoma, characterized primarily by hypogammaglobulinemia and B-cell deficiency. Patients with Good’s syndrome exhibit compromised immunity, rendering them highly susceptible to recurrent infections. This report describes the case of a patient who developed multiple episodes of pneumonia and persistent diarrhea following thymomectomy for thymoma, ultimately leading to the diagnosis of Good's syndrome. The clinical course highlights the importance of considering this diagnosis in patients with thymoma who present with recurrent infections.

## Introduction

Good's syndrome, first described by Dr Robert Good in 1955, is a rare acquired immunodeficiency disorder that occurs in association with thymoma. With an estimated prevalence of less than 0.5% among thymoma patients, this condition represents one of the least common primary immunodeficiencies in adults [1]. The syndrome is characterized by a triad of findings: thymoma, hypogammaglobulinemia, and cellular immune defects, leading to increased susceptibility to recurrent and opportunistic infections [2].

The diagnosis of Good's syndrome presents significant clinical challenges, as it is often delayed by several years following thymoma detection. Most patients come to medical attention only after developing recurrent infections, typically involving the respiratory and gastrointestinal systems [3]. Common pathogens include encapsulated bacteria such as *Streptococcus pneumoniae* and *Haemophilus influenzae*, as well as opportunistic organisms such as Salmonella species, Candida, and various viruses [1]. This pattern of infections reflects the combined humoral and cellular immune dysfunction that characterizes the condition.

Immunologically, Good's syndrome manifests with profound B-cell deficiency or complete absence, leading to hypogammaglobulinemia affecting all immunoglobulin classes. Cellular immunity is also impaired, with characteristic findings of CD4+ T-cell lymphopenia and abnormal T-cell proliferation responses. These defects persist despite thymomectomy, distinguishing Good's syndrome from other thymoma-associated autoimmune conditions where thymomectomy may be therapeutic [1,2]. Management primarily focuses on immunoglobulin replacement therapy to prevent infections, though the prognosis remains guarded. This case report highlights the importance of early recognition and intervention in this challenging condition.

## Case presentation

A 47-year-old man with no prior significant medical history was incidentally found to have an anterior mediastinal mass during imaging for COVID-19 pneumonia in June 2023. He subsequently underwent thymomectomy in December 2023, and the histopathological examination confirmed type AB thymoma.

Over the following year, he was admitted multiple times to different hospitals due to recurrent episodes of pneumonia, treated with several courses of antibiotics and antifungals. He also experienced chronic diarrhea during this time. In August 2024, he presented again with fever, fatigue, loose stools, and persistent cough. Blood investigations revealed anemia, with hemoglobin at 9.7 g/dL (normal range: 13.0-17.0 g/dL), a normal white cell count of 6.0 × 10⁹/L (normal: 4.0-11.0 × 10⁹/L), and mild thrombocytosis with platelets at 453 × 10⁹/L (normal: 150-400 × 10⁹/L). His C-reactive protein (CRP) was elevated at 184 mg/L (normal: <5 mg/L). Renal and liver function tests were within normal ranges. Infectious screening, including tests for tuberculosis, HIV, hepatitis B and C, and syphilis, was negative (Table 1).

**Table 1:** The patient's laboratory results.

Parameters	Patient values	Reference range
Hemoglobin	9.7 g/dL	13.0-17.0 g/dL
White cell count	6.0 × 10⁹/L	4.0-11.0 × 10⁹/L
Platelet	453 × 10⁹/L	150-400 × 10⁹/L
C-reactive protein	184 mg/L	<5 mg/L
Human immunodeficiency virus	Non-reactive	
Hepatitis B virus	Non-reactive	
Hepatitis C virus	Non-reactive	
Rapid plasma reagin	Non-reactive	
Immunoglobulin G	2.91 g/L	5.4-18.22 g/L
Immunoglobulin A	0.48 g/L	0.63-4.84 g/L
Immunoglobulin M	<0.15 g/L	0.22-2.40 g/L
B cells	0 × 10⁶/L	130-716 × 10⁶/L
Th cells (CD3+/CD4+ lymphocytes)	215 × 10⁶/L	431-1,976 × 10⁶/L
T cells (CD3+ lymphocytes)	1,034 × 10⁶/L	988-3912 × 10⁶/L
Tc cells (CD3+/CD8+ lymphocytes)	742 × 10⁶/L	385-1,805 × 10⁶/L
Natural killer cells	51 × 10⁶/L	227-1,354 × 10⁶/L

Stool culture initially revealed *Salmonella* spp., which was only susceptible to meropenem and resistant to ciprofloxacin, ceftriaxone, ampicillin, and co-trimoxazole. Intravenous meropenem was administered based on the susceptibility testing, but his diarrhea persisted. This prompted further stool microscopy testing, which confirmed the presence of *Microsporidium* spp., and he was subsequently started on oral albendazole for two weeks.

**Figure 1:**
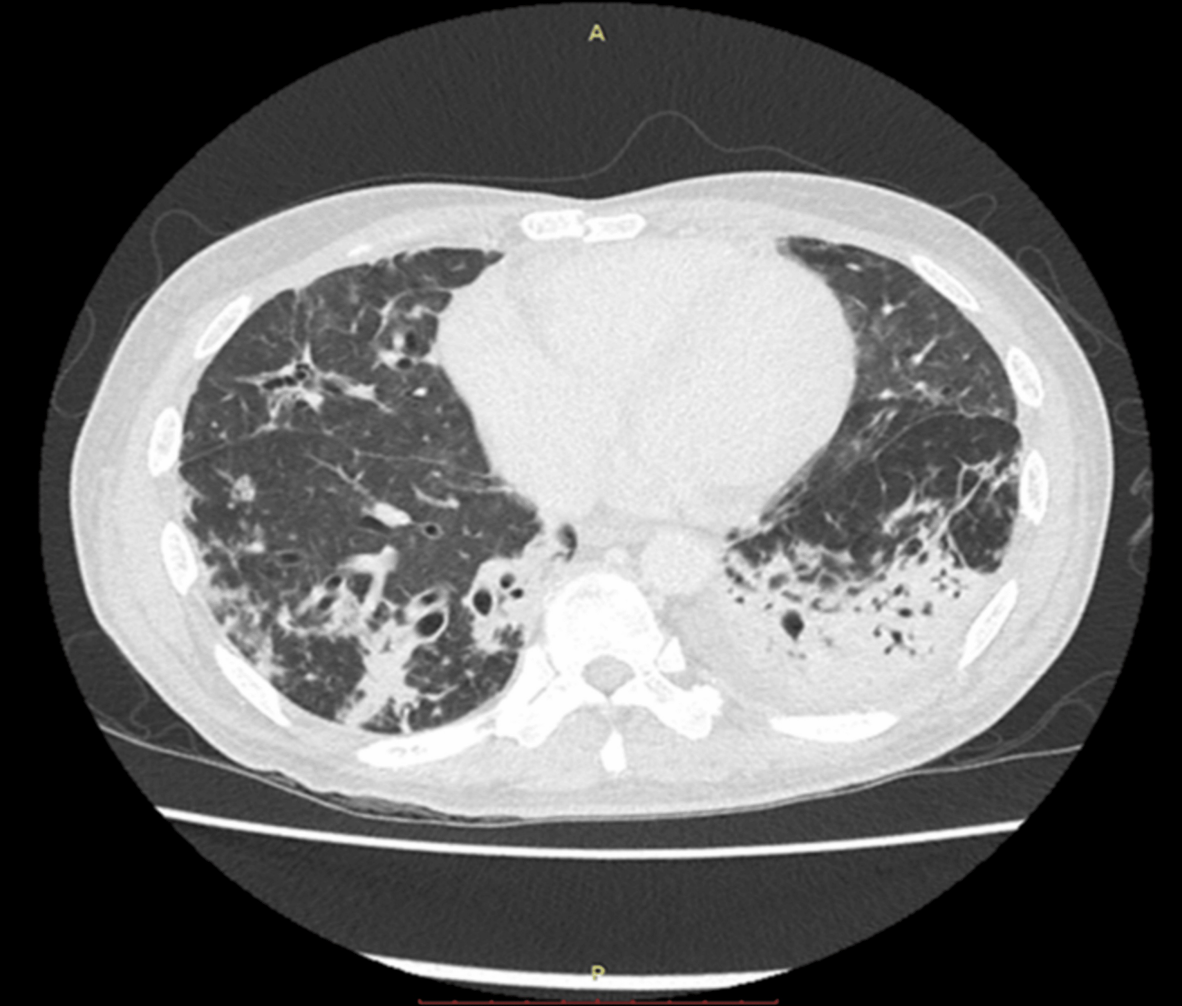
CT scan of the thorax showing consolidation with an air bronchogram in the posterior segment of bilateral lower lobes, as well as secondary organizing pneumonia, fibrosis, and bronchiectasis.

A computed tomography (CT) scan of the chest, abdomen, and pelvis demonstrated bilateral lower lobe consolidations with features of organizing pneumonia, fibrosis, and bronchiectasis (Figure 1). There were no abdominal or pelvic collections. These findings were consistent with chronic lung disease as a result of recurrent chest infections. Bronchoscopy showed purulent secretions in the left lower and right middle lobes. Bronchoalveolar lavage (BAL) fluid tested positive for galactomannan, with an index value of 2.7 (normal: <0.5), suggesting possible invasive fungal infection. However, fungal cultures and tests for *Mycobacterium tuberculosis* were negative. A colonoscopy revealed mild mucosal inflammation, and colonic biopsy showed active colitis with crypt architectural distortion, suggesting disease chronicity.

Given the history of recurrent respiratory and gastrointestinal infections, further evaluation for primary immunodeficiency was undertaken. Immunoglobulin levels were markedly reduced: IgG at 2.91 g/L (normal: 5.4-18.22 g/L), IgA at 0.48 g/L (normal: 0.63-4.84 g/L), and IgM at <0.15 g/L (normal: 0.22-2.40 g/L), confirming hypogammaglobulinemia. Flow cytometry revealed complete absence of circulating B cells (0 × 10⁶/L; normal: 130-716 × 10⁶/L), reduced Th cells (CD3+/CD4+ lymphocytes) at 215 × 10⁶/L (normal: 431-1,976 × 10⁶/L), and diminished natural killer (NK) cells at 51 × 10⁶/L (normal: 227-1,354 × 10⁶/L), while T cells (CD3+ lymphocytes) and Tc cells (CD3+/CD8+ lymphocytes) were normal at 1,034 × 10⁶/L (normal: 988-3912 × 10⁶/L) and 742 × 10⁶/L (normal: 385-1,805 × 10⁶/L), respectively (Table 1). These findings confirmed combined humoral and cellular immunodeficiency. The presence of thymoma, along with severe hypogammaglobulinemia and absence of B cells, was consistent with Good’s syndrome, a rare adult-onset immunodeficiency associated with thymoma.

During hospitalization, the patient received a single dose of intravenous immunoglobulin (IVIG) and was planned for regular monthly infusions. However, he requested early discharge and unfortunately passed away at home before receiving his next scheduled dose.

## Discussion

Good’s syndrome is a rare, adult-onset immunodeficiency associated with the presence of a thymoma. It is characterized by hypogammaglobulinemia, absence or severe reduction of peripheral B cells, and defects in cell-mediated immunity, including low CD4 T cell counts and an inverted or altered CD4/CD8 ratio [2,4]. While no standardized diagnostic criteria exist, the International Union of Immunological Societies classifies it under predominantly antibody deficiencies, recognizing the hallmark combination of thymoma and humoral immunodeficiency, often accompanied by T-cell abnormalities [5].

The majority of patients are diagnosed between the fourth and sixth decades of life, with symptoms typically developing before the thymoma is discovered. In a review, the average age at symptom onset was 56 years, with thymoma being diagnosed at around 62 years [6]. In contrast, our patient was diagnosed with thymoma at the age of 46, with immunodeficiency features such as low immunoglobulin levels and reduced CD4 and NK cell counts discovered about a year later.

Thymoma is frequently diagnosed months, if not years, before immunodeficiency or recurrent infections manifest [7,8]. In this case, the thymoma was discovered coincidentally during a COVID-19 admission, and it took nearly 15 months to confirm the diagnosis of Good's syndrome. Although Good's syndrome has been linked to autoimmune disorders such as myasthenia gravis, pure red cell aplasia, pernicious anemia, and autoimmune thrombocytopenia, our patient had no autoimmune symptoms, and autoimmune screening tests were negative [9].

The clinical course of Good’s syndrome is often dominated by recurrent infections, particularly affecting the respiratory and gastrointestinal systems [2,6]. Pulmonary infections are common and frequently lead to bronchiectasis over time due to repeated inflammation and tissue damage. This patient experienced multiple hospital admissions for pneumonia, requiring numerous courses of antibiotics and antifungals. Additionally, he suffered from chronic diarrhea, with stool testing positive for *Salmonella* spp. and *Microsporidium* spp., both opportunistic pathogens in immunocompromised hosts.

The pathophysiological mechanism linking thymoma and immunodeficiency in Good’s syndrome remains poorly understood. Although thymomectomy is the standard treatment for thymoma and other thymoma-associated autoimmune diseases, it does not improve the immunological abnormalities associated with Good’s syndrome [4,10]. In our case, the patient's immunodeficiency persisted following thymomectomy.

Immunoglobulin replacement therapy is considered the mainstay of treatment for infection control in Good’s syndrome. It has been associated with a reduction in the frequency and severity of bacterial infections [11,12]. Our patient received a single dose of IVIG during his final hospitalization but unfortunately passed away at home before further doses could be administered. As previously reported, IVIG dosing typically ranges from 300 to 500 mg/kg every three to four weeks [13]. Despite this, outcomes in Good’s syndrome tend to be less favorable compared to other immunodeficiency syndromes [12].

## Conclusions

Clinicians should maintain a high index of suspicion for Good’s syndrome in adults with thymoma and unexplained recurrent infections. The key immunological features include severe hypogammaglobulinemia and profound B-cell depletion, often accompanied by T-cell and NK cell deficiencies. Early recognition and long-term immunoglobulin replacement therapy are essential to improve patient outcomes. However, despite treatment, the prognosis remains guarded, with infections being the leading cause of morbidity and mortality.
